# All PI3Kinase signaling is not mTOR: dissecting mTOR-dependent and independent signaling pathways in T cells

**DOI:** 10.3389/fimmu.2012.00312

**Published:** 2012-10-09

**Authors:** Christopher J. Gamper, Jonathan D. Powell

**Affiliations:** Department of Oncology, Johns Hopkins UniversityBaltimore, MD, USA

**Keywords:** mTOR pathway, PI3K, CD4 T cells, effector function, tolerance

## Abstract

The mechanistic target of rapamycin (mTOR) is emerging as playing a central role in regulating T cell activation, differentiation, and function. mTOR integrates diverse signals from the immune microenvironment to shape the outcome of T cell receptor (TCR) antigen recognition. Phosphatidylinositol 3-kinase (PI3K) enzymes are critical mediators of T cell activation through their generation of the second messenger phosphatidylinositol (3,4,5) triphosphate (PIP3). Indeed, PIP3 generation results in the activation of Protein Kinase B (PKB, also known as AKT), a key activator of mTOR. However, recent genetic studies have demonstrated inconsistencies between PI3K disruption and loss of mTOR expression with regard to the regulation of effector and regulatory T cell homeostasis and function. In this review, we focus on how PI3K activation directs mature CD4 T cell activation and effector function by pathways dependent on and independent of mTOR signaling. Importantly, what has become clear is that targeting both mTOR-dependent and mTOR-independent PI3K-induced signaling distally affords the opportunity for more selective regulation of T cell differentiation and function.

## Introduction

The Class IA phosphatidylinositol 3-kinase (PI3K) family consists of a heterodimeric complex of one of the 110-kDa catalytic subunits (p110α, β, δ) with a regulatory subunit (p85α, p55α, p50α, p85β, and p55γ), reviewed in Kane and Weiss (2003); Okkenhaug and Vanhaesebroeck (2003). Catalytic subunits are each encoded by separate genes, while the *pik3r1* gene generates p85α, p55α, and p50α from alternative promoters and the *pik3r2* and *pik3r3* genes encode p85β and p55γ, respectively. Details of receptor interactions with PI3Ks have recently been reviewed elsewhere (Okkenhaug and Fruman, 2010). Co-localization of catalytic subunits with regulatory subunits permits juxtaposition of catalytic subunits to the cell membrane in response to receptor ligation, increasing the local synthesis of phosphatidylinositol (3,4,5) triphosphate (PIP3). The p110α and β subunits are ubiquitously expressed while p110δ expression is restricted to hematopoietic cells. The Class IB PI3 Kinase family consists of a complex of the p110γ catalytic subunit and either the p101 or p84 regulatory subunits. This complex interacts with G-protein coupled receptors including chemokine receptors via binding to Gβ and γ regulatory proteins. Like p110δ, p110γ expression is restricted to hematopoietic cells. Accumulation of the PIP3 signaling intermediate is opposed by phosphatase and tensin homolog (PTEN) that converts PIP3 back to phosphatidylinositol (4,5) bisphosphate and Src homology 2 domain-containing inositol phosphatase (SHIP)1 and SHIP2 that hydrolyze PIP3 to phosphatidylinositol (3,4) bisphosphate (Okkenhaug and Fruman, 2010).

PIP3 acts as a second messenger to mediate downstream signaling by recruitment of pleckstrin homology (PH) domain containing proteins that bind to the high local concentrations of PIP3 generated by PI3Ks at the inner leaflet of the plasma membrane. Examples include the Tec family of tyrosine kinases that mediate signals to phospholipase C-γ (PLCγ), the 3-phosphoinositide-dependent protein kinase 1 (PDK1), and Protein Kinase B (PKB), also known as AKT [reviewed in Kane and Weiss (2003)]. Members of the Vav family of guanine nucleotide exchange factors that regulate cellular motility may also be recruited via PIP3 binding, although some data suggest recruitment may be indirect via other protein interactions. AKT binding to PIP3 induces a conformational change that renders it accessible to phosphorylation at residue T308 by PDK1 co-localized at the plasma membrane, resulting in activation of AKT serine/threonine kinase activity (Stokoe et al., 1997; Currie et al., 1999; Milburn et al., 2003). One critical substrate for activated AKT is tuberous sclerosis complex (TSC)-2. TSC2 functions with TSC1 as a GTPase activating complex for Ras homolog enriched in brain (Rheb). TSC2 is inactivated by phosphorylation, resulting in accumulation of GTP-bound Rheb that activates the mechanistic target of rapamycin (mTOR) that is in a complex with regulatory-associated protein of mTOR (Raptor) termed mTORC1. Activated mTORC1 phosphorylates and inhibits the eukaryotic initiation factor 4E-binding proteins (4E-BP1, 2, 3) and activates the p70 ribosomal S6 kinases (S6K1, 2), resulting in increased protein translation and upregulation of glycolysis, promoting cell growth and division [reviewed in Laplante and Sabatini (2012)]. In addition, mTORC1 activation is required for several key elements of T cell effector differentiation that are discussed in further detail below.

A second mTOR complex containing the rapamycin insensitive companion of TOR (Rictor) and the mammalian homolog of the yeast *SIN1* gene (mSIN1), termed mTORC2, is also critical to mediating PI3K signaling via AKT. Although the precise mechanisms underlying activation of mTORC2 remain incompletely understood, it was recently observed that physical association with ribosomes stimulates mTORC2 activity (Zinzalla et al., 2011) and that Rictor acetylation by p300 stimulates mTORC2 activity (Glidden et al., 2012). mTORC2 is strongly activated in T cells by costimulation and cytokines [reviewed in Cantrell (2002)]. mTORC2 has several substrates including serum- and glucocorticoid-induced protein kinase 1 (SGK1), protein kinase C-α (PKC-α), and importantly, AKT itself. Phosphorylation at residue S473 in the AKT hydrophobic motif modifies substrate specificity of AKT and enhances its kinase activity (Sarbassov et al., 2005; Facchinetti et al., 2008; Garcia-Martinez and Alessi, 2008). In particular, S473 phosphorylation is required to permit AKT to phosphorylate members of the Forkhead box family of transcription factors Foxo1 and Foxo3a (Jacinto et al., 2006). Foxo phosphorylation leads to the cytoplasmic retention of these transcription factors with resulting downregulation of target gene expression (Brownawell et al., 2001). In T cells, a key Foxo target is KLF2 that regulates expression of CD62L, the c–c chemokine receptor 7 (CCR7), and the sphingosine-1-phosphate receptor (S1P1R) which each control homing of lymphocytes into and out of secondary lymphoid tissues [reviewed in Finlay and Cantrell (2010)].

The signaling cascade from receptors through PI3K to mTOR described above appears to imply a linear relationship, and older studies utilizing small molecule inhibitors of either PI3Ks such as wortmannin or LY294002 or mTOR using rapamycin demonstrated similar profound inhibition of T cell function *in vitro* (Kay et al., 1991; Ward et al., 1995). However, recent analysis of the pathways using more specific inhibitors and genetic manipulation has identified a more complex signaling cascade with both overlapping and unique functions. Phenotypes of mice discussed in this review with targeted disruption of components of the PI3K or mTOR pathways are summarized in Table 1 below.

**Table 1:** **T cell phenotype and function in PI3K and mTOR pathway gene-targeted mice**.

**Gene**	**Phenotype**	**References**
*pik3r1* (p85α^−/−^, p55α^−/−^, p50α^−/−^)	Normal T cell development and proliferation	Fruman et al., 1999
*pik3r1* (p85α^−/−^)	Normal T cell development and proliferation	Suzuki et al., 1999
*pik3r2* (p85β^−/−^)	Normal T cell development	Deane et al., 2004
	Enhanced proliferation to TCR stimulation or TCR + IL-2	
r1ΔT/r2n (p85α^−/−^, p55α^−/−^, p50α^−/−^, p85β^−/−^)	Normal T cell development	Oak et al., 2006
	Normal Th1 but reduced Th2 differentiation	Deane et al., 2007
	Impaired T-dependent antibody response	
	Reduced Treg numbers, spontaneous autoimmunity	
*pik3cd* (p110δ^−/−^, CD4-Cre;p110δ^flox/flox^, or OX40-Cre;p110δ^flox/flox^)	Normal T cell development and proliferation	Clayton et al., 2002
	Impaired T-dependent antibody response	Rolf et al., 2010
*pik3cd* (p110δ^D910A/D910A^)	Decreased peripheral T cell numbers	Okkenhaug et al., 2002
	CD3/CD28-induced proliferation and IL-2 normal	Okkenhaug et al., 2006
	Antigen-induced proliferation and IL-2 reduced	Patton et al., 2006
	Impaired Th1 and Th2 differentiation *in vitro*	Nashed et al., 2007
	Reduced Th1 contact hypersensitivity	Liu et al., 2009
	Inappropriate Th1 differentiation to Th2 stimulus *in vivo*	Rolf et al., 2010
	Reduced Th17 differentiation with attenuated EAE	Soond et al., 2010
	Reduced IL-10 production	Haylock-Jacobs et al., 2011
	Impaired Treg function with autoimmune colitis	
	Impaired T-dependent antibody response	
*pik3cg* (p110γ^−/−^)	Normal T cell development	Sasaki et al., 2000
	Variably decreased or normal proliferation	Alcazar et al., 2007
	Variably decreased or normal cytokine production	Garcon et al., 2008
	Abnormal trafficking to sites of inflammation	Martin et al., 2008
	Impaired trafficking to chemokines	Thomas et al., 2008
*pten* (Lck-Cre;PTEN^flox/−^ or OX40-Cre;PTEN^flox/flox^)	Lymphoproliferative disorder (Lck-Cre)	Suzuki et al., 2001
	Enhanced T cell proliferation and cytokine production	Soond et al., 2012
	Spontaneous autoimmunity (Lck-Cre)	
	Enhanced T cell help (OX40-Cre)	
	Enhanced tumor rejection (OX40-Cre)	
*frap1* (CD4-Cre;mTOR^flox/flox^)	Normal T cell development	Delgoffe et al., 2009
	Reduced proliferation	
	Normal initial IL-2 secretion	
	Impaired Th1, Th2, and Th17 differentiation	
	Spontaneous iTreg differentiation	
*rheb* (CD4-cre;rheb^flox/flox^)	Normal T cell development	Delgoffe et al., 2011
	Impaired Th1 and Th17 differentiation	
	Normal Th2 and iTreg differentiation	
*raptor* (Lck-Cre;raptor^flox/flox^)	Reduced peripheral T cell numbers	Kurebayashi et al., 2012
	Impaired Th17 differentiation	
*rictor* (CD4-Cre;rictor^flox/flox^ or proximal Lck-Cre;rictor^flox/flox^)	Normal T cell development	Lee et al., 2010
	Variably decreased Th1 and Th17 differentiation	Delgoffe et al., 2011
	Impaired Th2 differentiation	
	Normal iTreg differentiation	
	Impaired T-dependent antibody response	

## Interruption of the PI3K/AKT/mTOR pathway at different levels has differential effects on T cell activation

A critical aspect to TCR signaling is its ability to activate PLCγ and generate the second messenger inositol triphosphate (IP3) that triggers Ca^++^ entry following activation. This Ca^++^ binds calmodulin and activates calcineurin, which dephosphorylates NF-AT transcription factors that translocate to the nucleus to activate transcription of genes that regulate both T cell activation/effector function and tolerance (Macian et al., 2002). Cyclosporine acts as a potent immunosuppressant by binding to cyclophilin and blocking activation of calcineurin and thus NF-AT. Rapamycin inhibits T cell activation by a different mechanism, by binding to FKBP12 and preventing association of Raptor and mTOR to form the mTORC1 complex. The importance of this difference is highlighted by the fact that activation of Th1 cells in the presence of rapamycin renders them subsequently anergic and incapable of proliferation and IL-2 secretion upon secondary stimulation after rapamycin is washed away, in a manner similar to Th1 cells stimulated through the T cell receptor (Signal 1) in the absence of costimulation via CD28 (Signal 2) (Powell et al., 1999). This occurs despite the fact that while rapamycin acutely inhibits T cell proliferation, it does not block initial IL-2 secretion. Cyclosporine, in contrast, blocks both proliferation and IL-2 secretion acutely but does not impair secondary stimulation after drug is removed. Rapamycin appears to promote anergy and tolerance by permitting normal TCR-mediated activation of *NF-AT* gene targets including the transcription factors Egr-2 and Egr-3 and their targets such as Cbl-b, DGKα, and Fas-L that limit T cell activation and promote apoptosis (Sanjuan et al., 2001; Macian et al., 2002; Safford et al., 2005; Zha et al., 2006). As these signaling pathways would predict, co-incubation of T cells with cyclosporine and rapamycin blocks rapamycin-induced anergy (Powell et al., 1999). That is, blocking proximal TCR-mediated signaling with cyclosporine mitigates the effects of blocking mTOR with rapamycin. We emphasize the consequences of blocking proximal signaling with cyclosporine and more distal signaling with rapamycin because a parallel can be drawn comparing genetic blockade of mTOR that promotes generation of regulatory T cells (Treg) and genetic blockade of proximal PI3K signaling that results in decreased Treg function *in vivo* (to be discussed in depth below).

The generation of mice with T cell conditional deletion of mTOR using a floxed allele crossed with a CD4-Cre transgenic mouse confirms the specificity of rapamycin for blocking mTOR-mediated signaling (Delgoffe et al., 2009). Stimulation of mTOR deficient T cells with anti CD3/CD28 induces robust, even slightly increased, proximal AKT T308 phosphorylation but it is unable to elicit S6K1 and AKT S473 phosphorylation. Like rapamycin treated T cells, mTOR deficient T cells show normal IL-2 secretion during primary stimulation but reduced proliferation. However, the consequence of activation of naïve T cells lacking mTOR is the inhibition of effector differentiation and the generation of Tregs.

Since PI3K is upstream of mTOR, the effect of a PI3K inhibitor would be predicted to mimic rapamycin. In fact, stimulation of T cells in the presence of wortmannin does acutely block proliferation, but IL-2 secretion is also inhibited, similar to cyclosporine (Ward et al., 1995). Despite this difference, the presence of wortmannin during T cell priming does result in anergy upon restimulation similar in magnitude to that achieved by costimulatory blockade with CTLA4-Ig which blocks B7/CD28 interactions (Taub et al., 1997). Importantly, subsequent work has demonstrated that wortmannin is not selective for PI3K as originally thought, but it can inhibit mTOR as well (Brunn et al., 1996), therefore it is not clear if the findings reported by Taub et al. merely mimic the effect of rapamycin-induced mTOR blockade. Clarification of distinctions between PI3K and mTOR-dependent signals has subsequently been provided by genetic analysis.

Initial work deleting individual PI3K regulatory subunits demonstrated significant functional redundancy in T cells, with no overt defects in T cell development and signaling after disruption of p85α and paradoxically enhanced TCR and IL-2-induced proliferation in p85β knockouts (Fruman et al., 1999; Suzuki et al., 1999; Deane et al., 2004); therefore the p85α and p85β knockouts were crossed to disrupt all Class IA PI3K activity. This was accomplished by crossing *pik3r1*^2loxP/2loxP^ mice with *pik3r2*^−/−^ mice and breeding these onto an Lck-Cre background to cause T cell specific deletion of both regulatory subunits (Deane et al., 2007). Resulting mice, termed r1ΔT/r2n, have normal generation of T cells, but such cells have profound decreases in expression of multiple PI3K catalytic subunits with greatly reduced p110β and undetectable p110α and p110δ. Such T cells demonstrate expected loss of CD3/CD28-induced AKT S473 phosphorylation. This may be due to impaired mTORC2 signaling but it is important to comment that recruitment of AKT to the cell membrane to be phosphorylated by PDK1 at T308 is predicted to be interrupted as well. Since T308 phosphorylation was not directly measured, it cannot formally be determined whether the loss of AKT S473 phosphorylation represents a lack of singly phosphorylated AKT substrate for mTORC2 or is a consequence of reduced mTORC2 activity. Also, phosphorylation of other mTORC2 substrates such as SGK1 and PKCα was not reported in this study. Similar data on AKT S473 phosphorylation in isolation without other measures of mTORC2 activity has been extensively reported in other systems described in this review. Thus, interpretation of relative levels of mTORC2 activity in some of these systems remains speculative but is helpful to consider when comparing phenotypes with systems where mTORC2 is selectively disrupted. Deane et al. unexpectedly provide evidence for preservation of some CD3/CD28-induced phosphorylation of ribosomal S6 at S235/236. While some data have suggested that this particular S6 phosphorylation site may be more dependent on RSK activity than mTOR activity upstream of S6K (Salmond et al., 2009), Deane et al. demonstrate that this S6 phosphorylation in r1ΔT/r2n T cells is blocked by rapamycin, supporting that in r1ΔT/r2n T cells some mTORC1 activity is preserved. TCR-induced calcium flux was modestly reduced in r1ΔT/r2n T cells and TCR-induced NF-κ B activation as measured by Iκ B phosphorylation was significantly reduced, consistent with reduced activation of the Tec kinase/PLCγ pathway due to inefficient PIP3 generation. However, Iκ B phosphorylation could be normalized with CD28 costimulation. This partial preservation of CD3/CD28-induced signals including some mTOR activation may explain why proliferation is only slightly reduced while IL-2 and IFN-γ are more profoundly suppressed, which would not be predicted by the PI3K and rapamycin inhibitor studies described above and differs from the phenotype following T cell specific deletion of mTOR.

Since disruption of the regulatory subunits indirectly affects expression of multiple PI3K catalytic subunits, targeted deletion of individual catalytic subunits has been used to dissect how each one contributes to T cell activation with the rationale that a selective inhibitor of a T cell dominant PI3K isoform might have less off target effects in non-immune cells and be a more useful immunosuppressant. Two parallel approaches have been used to determine the role of the PI3K p110δ isoform in lymphocytes, simple gene deletion and targeted knock-in of a kinase inactive mutant p110δ D910A allele (Clayton et al., 2002; Okkenhaug et al., 2002). The latter mice were generated due to concern that deletion of individual regulatory or catalytic subunits appears to result in compensatory changes in expression of remaining *PI3K* genes. Also, the catalytically inactive protein could still function in a scaffolding capacity during assembly of receptor-induced signaling complexes (Okkenhaug et al., 2002). p110δ^−/−^ mice (completely deleted for p110δ) have normal peripheral T cell numbers but impaired T-dependent antibody production (Clayton et al., 2002). Subsequent work relying on T and B cell conditional deletion of a p110δ floxed allele demonstrated that loss of p110δ expression in T cells due to conditional deletion with Lck-Cre is sufficient to disrupt T-dependent antibody responses despite preserving essentially normal T cell numbers and normal T cell proliferation in response to CD3/CD28 stimulation. This is correlated with an absence of AKT S473 phosphorylation in response to stimulation with anti CD3 plus ICOS-L and significantly decreased expression of CD40L by activated p110δ^−/−−^ T cells (Rolf et al., 2010). The p110δ^D910A/D910A^ mice demonstrate normal cellularity of the thymus but have about a 50% reduction in mature T cell numbers and a decreased frequency of CD44^high^ effector T cells in the periphery. Phosphorylation of AKT on S473 is severely reduced after CD3 crosslinking, however T cell proliferation and IL-2 secretion when stimulated with anti CD3/CD28 coated beads was the same as WT mice. This stands in contrast to the diminished proliferation and IL-2 secretion that was seen when p110δ^D910A/D910A^ were crossed to a TCR Tg and stimulated with cognate peptide/APC, indicating that strength of stimulation may compensate in some manner for loss of efficient PIP3 generation in such cells, perhaps via activation of p110γ as discussed below (Alcazar et al., 2007). Similar to r1ΔT/r2n mice, mTOR-independent TCR signaling to induce NF-κ B activation was reduced in p110δ^D910A/D910A^ T cells but NF-κ B activation normalized when CD28 costimulation was provided (Okkenhaug et al., 2006). In summary, loss of the individual p110δ subunit or all class IA PI3Ks results in consistent inhibition of AKT S473 phosphorylation implying decreased mTORC2 activity, but TCR-induced proliferation may be relatively spared, depending on the strength of the TCR stimulation. Loss of IL-2 production also appeared to be more severe in the absence of all Class IA PI3K expression. These effects differ from the effects of rapamycin treatment or deletion of mTOR where proliferation is more severely inhibited, initial IL-2 production is preserved, but recall cytokine responses are diminished. Mechanisms underlying these differences may relate to CD4 T cell effector differentiation and are discussed in the next section.

There are conflicting data on the coupling of p110γ to TCR signaling. In one report, T cells from p110γ^−/−^ mice have reduced anti CD3 and anti CD3/CD28-induced proliferation, IL-2 production, and IFN-γ production (Sasaki et al., 2000), to some extent resembling T cell anergy. However, while the addition of exogenous IL-2 can rescue Th1 cell anergy induced by rapamycin (Powell et al., 1999), supplementation of IL-2 only partially corrects the defective proliferation of p110γ^−/−^ T cells in response to anti CD3/CD28 (Alcazar et al., 2007). Furthermore, p110γ^−/−^ T cells demonstrate reduced AKT S473 and mitogen activated protein kinase (MAPK) phosphorylation after stimulation with anti CD3 or anti CD3/CD28, correlating with decreased F-actin polymerization and impaired formation of stable conjugates between T cells and peptide loaded APCs (Alcazar et al., 2007). These data stand in contrast to studies which examined early signaling events in p110γ^−/−^ T cells following TCR binding to cognate peptide on APC. Recruitment of an AKT PH domain-GFP fusion protein to the immunologic synapse following stimulation with peptide loaded APC is normal in T cells lacking p110γ while it is impaired in p110δ^D910A/D910A^ T cells and CD28^−/−^ T cells (Garcon et al., 2008). Likewise, proliferation, downregulation of CD62L, and upregulation of CD44 in response to antigen *in vivo* is normal in CD4 T cells lacking p110γ (Thomas et al., 2008). Proliferation of CD8 T cells lacking p110γ was also normal in response to antigen pulsed APC *in vitro* and vaccinia virus expressing a model antigen *in vivo* (Martin et al., 2008). The mechanism underlying differences between the normal proliferation to antigen but reduced proliferation to anti CD3/CD28 crosslinking in p110γ^−/−^ T cells as compared to the reduced proliferation to antigen but near normal proliferation to anti CD3/CD28 in T cells lacking normal p110δ remains incompletely understood, but it may be related to increased susceptibility of p110γ^−/−^ T cells to activation induced cell death as p110γ^−/−^ thymocytes showed enhanced death compared to WT when exposed to anti CD3 in the presence of adenosine receptor agonists (Sasaki et al., 2000). It will be interesting to see if preservation of antigen induced mTOR activation contributes to the proliferation seen from p110γ^−/−^ T cells.

Deletion of PTEN in tumors is associated with constitutive accumulation of PIP3 and appears to promote proliferation and tumor survival, thus it was not surprising when mice with T cell conditional deletion of PTEN were found to develop a lymphoproliferative disorder and die prematurely of CD4 T cell lymphomas (Suzuki et al., 2001). Prior to the onset of lymphoma, peripheral T cells from such mice demonstrate enhanced proliferation, IL-2 secretion, IFN-γ secretion, and AKT S473 phosphorylation after CD3/CD28 stimulation. T cells demonstrate a spontaneous activated, CD69 high phenotype and are autoreactive. Furthermore, such mice develop autoimmunity and hypergammaglobulinemia due to aberrant self-tolerance. This immunopathology appears strongly related to the timing of loss of PTEN expression because conditional deletion of PTEN after completion of thymic negative selection using OX40-Cre, which results in deletion in mature peripheral T cells after initial activation, does not lead to development of autoreactive cells or systemic autoimmunity despite increased lymph node cellularity (Soond et al., 2012). OX40-Cre PTEN null T cells do exhibit enhanced CD3-induced proliferation and IFN-γ production compare to WT. Furthermore, consistent with an exaggerated AKT/mTOR activation state, such mice develop an increased secondary contact hypersensitivity recall response.

## Naïve CD4 T cells require PI3K-induced mTOR-dependent and mTOR-independent signals

Naïve CD4 T cells acquire restricted patterns of cytokine expression or regulatory function as a consequence of cytokine exposure or strength of antigenic stimulation during the period of initial activation. Specific patterns of cytokine expression are associated with master transcriptional regulators in the different lineages: Th1 cells express Tbet and IFN-γ ; Th2 cells express GATA3 and IL-4, IL-5, and IL-13; Th17 cells express ROR-γ T and IL-17A/F; Treg express FoxP3 and suppress other T cell responses [reviewed in Weaver et al. (2006)]. More recently an additional subset termed T follicular helper (Tfh) cell was identified that supports generation of T-dependent antibody production and is dependent on expression of the transcription factor Bcl6 and marked by surface ICOS and CXCR5 and secretion of IL-21 [reviewed in Crotty (2011)]. A requirement for mTOR activation in helper T cell differentiation was initially suggested by the observation that rapamycin inhibition of mTOR promotes *de novo* expression of FoxP3 by naïve T cells and promotes expansion of Tregs *in vitro* (Battaglia et al., 2005). Such rapamycin-induced FoxP3+ cells are functional Tregs capable of suppressing pancreatic allograft rejection *in vivo*. Additional evidence of a critical role for mTOR signals in promoting effector function and inhibiting Treg generation comes from the mice with conditional deletion of mTOR in T cells. Following initial activation, a high proportion of mTOR deficient T cells spontaneously become inducible FoxP3+ Tregs and Th effector differentiation to Th1, Th2, and Th17 fates *in vitro* is severely impaired (Delgoffe et al., 2009).

Mice with individual T cell conditional deletion of either mTORC1 or mTORC2 have provided surprising insight into non-overlapping roles for each of these complexes in normal T cell effector differentiation. Selective loss of mTORC1 following Rheb deletion results in CD4 T cells incapable of Th1 and Th17 differentiation but preserves the ability to make Th2 cells in response to IL-4 stimulation (Delgoffe et al., 2011). More recently, T cell conditional deletion of Raptor was shown to impair Th17 differentiation but Th1 differentiation was preserved (Kurebayashi et al., 2012). Conversely, deletion of Rictor with resulting loss of mTORC2 causes loss of Th2 differentiation, with variable effects on Th1 and Th17 differentiation depending on the timing of Cre expression in the thymus based on the particular Cre transgenic line that the floxed Rictor allele is crossed to (Lee et al., 2010; Delgoffe et al., 2011). T cell-dependent antibody production in mice with mTORC1 deficient T cells is normal but mice with mTORC2 deficient T cells have decreased antibody titers in both mouse lines, although enumeration of Tfh was not performed. As Th17 cells contribute significantly to the pathology of a murine model for multiple sclerosis, experimental autoimmune encephalitis (EAE), the Rheb deficient T cell mice were compared to WT in the EAE system. Rheb deficiency in T cells results in lower EAE severity scores than WT despite all mice developing some symptoms. This correlates with fewer T cells infiltrating the spinal cord and a lower frequency of IL-17 and IFN-γ secretion by T cells extracted from spinal cords of the Rheb deficient T cell mice. Strikingly, 60% of the Rheb deficient T cell mice immunized to induce EAE develop ataxia consistent with “non-classical EAE” that has been observed with induction of Th2 cells in other reports (Wensky et al., 2005). Rheb deficient mice with ataxia have T cell infiltrates in their cerebellum that produce Th2 cytokines *in vitro* in response to antigen (Delgoffe et al., 2011). Treg number and function are normal following T cell conditional deletion of Rheb and Rictor, but Rictor deficient T cells that only have mTORC1 demonstrate enhanced sensitivity to rapamycin in promoting expression of FoxP3 *in vitro* (Delgoffe et al., 2011). The observation that prolonged incubation of cells with higher doses of rapamycin blocks mTORC2 assembly serves to explain both this enhanced sensitivity of Rictor deficient T cells to rapamycin and the fact that loss of both mTORC1 and mTORC2 by deletion of mTOR is necessary to mimic the effect of rapamycin on promoting Tregs (Sarbassov et al., 2006).

Based on the regulation of mTOR by PI3K, the PI3K knockout models should recapitulate the effects of mTOR deletion on effector differentiation. However, just as the mTORC1 and mTORC2 deficient T cells demonstrated unexpected effector phenotypes, disruption of PI3K proximal signaling has also provided surprises. T cells from the r1ΔT/r2n mice lacking all p85 regulatory subunits and having low or absent p110α, β, and δ catalytic subunit expression supported normal Th1 polarization but Th2 cultures had decreased IL-4 and increased IFN-γ. This is consistent with the report above from Delgoffe et al. noting selective loss of IL-4 expression from T cells deficient in mTORC2 and the relative preservation of mTORC1-dependent S6 phosphorylation over mTORC2-dependent AKT S473 phosphorylation that Deane et al. report in the r1ΔT/r2n mice. However, the frequency of CD4^+^CD25^+^FoxP3^+^ natural Tregs in the spleen was significantly decreased in r1ΔT/r2n mice despite normal thymic Treg numbers. This is in contrast to normal Treg numbers in the periphery of mice with T cells lacking Rictor (Lee et al., 2010; Delgoffe et al., 2011). T-dependent B cell responses to NP-Ova were impaired and germinal centers following vaccination were decreased but antiviral responses to mouse hepatitis virus were normal (Deane et al., 2007). In addition, the mice were noted to develop spontaneous autoimmunity with corneal opacities and eye lesions found to resemble Sjögren's syndrome. On necropsy the r1ΔT/r2n mice were noted to have a predominantly CD4 T cell infiltrate into lacrimal and salivary lands with lymphadenopathy, splenomegaly, and anti-nuclear and anti SSA autoantibodies (Oak et al., 2006). A mechanistic link between decreased Tregs and autoimmunity was speculated upon but not formally tested in this report. These clinical findings arise in the mice despite the biochemical profile with loss of AKT S473 phosphorylation that resembles the Rictor deficient T cell mice that lack autoimmunity. This serves to highlight the importance of additional PI3K signaling pathways distinct from the AKT/mTOR axis in the maintenance and function of Tregs.

Further characterization of the p110δ^D910A/D910A^ mice demonstrated that Th1 and Th2 differentiation *in vitro* is markedly reduced (Okkenhaug et al., 2006). Interestingly, when *in vivo* Th2 differentiation was examined following immunization with Ova/Alum, p110δ^D910A/D910A^ mice exhibit decreased Th2 responses and inappropriate Th1 responses. That is, splenocytes have decreased Ova specific IL-4, IL-5, and IL-13 secretion but enhanced Ova specific IFN-γ and CXCL10 secretion, and the mice demonstrate a reduction in Th2-dependent allergic airway inflammation *in vivo*. This appears to be secondary to defective IL-10 production by p110δ^D910A/D910A^ T cells because neutralization of IL-10 *in vitro* during Ova stimulation enhances WT splenocyte IFN-γ production and addition of IL-10 suppresses p110δ^D910A/D910A^ splenocyte IFN-γ production (Nashed et al., 2007). Phosphorylation of mTORC1 substrates is not described in these manuscripts to see if the phenotype is related to imbalance of mTORC1 versus mTORC2, as is ostensibly predicted by the Rictor conditional deletion data above.

Th17 differentiation is also partially p110δ-dependent. IL-17A production from Th17 culture *in vitro* is strongly inhibited by the p110δ isoform specific inhibitor IC87114 while IFN-γ production in Th1 culture is only modestly decreased. Likewise, induction of Th17 cells in p110δ^D910A/D910A^ mice *in vivo* in EAE is decreased while Th1 responses are spared, resulting in less severe clinical symptoms (Haylock-Jacobs et al., 2011). This preservation of Th1 cytokine expression resembles the phenotype noted for the r1ΔT/r2n mice and to some degree the Rictor T cell deficient mice noted above (Deane et al., 2007; Delgoffe et al., 2011).

Despite the preferential Th1 phenotype under a typical Th2 stimulus and in the EAE model above, Th1-driven contact hypersensitivity responses in p110δ^D910A/D910A^ mice are reduced (Soond et al., 2010). This would lead to a prediction that such mice would have increased susceptibly to the Th1-sensitive pathogen *Leishmania major*. However, while p110δ^D910A/D910A^ mice have diminished T cell proliferation, IFN-γ, and TNF-α production to *Leishmania* antigens after infection, consistent with impaired Th1 function, the clinical outcome of infectious challenge is actually enhanced resistance, smaller lesion size, and more rapid parasite clearance. This was found to correlate with impaired generation of IL-10 producing Treg and recruitment of such Tregs to sites of infection in p110δ^D910A/D910A^ mice (Liu et al., 2009). Adoptive transfer of WT Tregs to p110δ^D910A/D910A^ mice reverses their *Leishmania* resistance, supporting the hypothesis that Treg dysfunction permits the inefficient p110δ^D910A/D910A^ Th1 response to be sufficient to clear infection. Additional evidence for functional Treg deficiency in the p110δ^D910A/D910A^ mice comes from their spontaneous development of autoimmune colitis. Such mice have 50% reduction in Treg frequency in the periphery compared to WT and no detectable IL-10 secretion by cultured Tregs. This is associated with reduced *in vitro* suppression compared to WT Tregs and an inability of p110δ^D910A/D910A^ Tregs to suppress colitis induced by adoptive transfer of naïve T cells into RAG1^−/−^ mice (Patton et al., 2006). In contrast to these autoimmune findings in mice with proximal PI3K defects, to date, no spontaneous autoimmune phenotype has been reported in mice with T cell conditional disruption of mTORC1, mTORC2, or both (Delgoffe et al., 2009, 2011; Lee et al., 2010).

Reminiscent of the mice with T cells lacking mTORC2 complexes and the r1ΔT/r2n mice, p110δ^D910A/D910A^ mice demonstrate abnormal T-dependent antibody responses. Such mice are largely devoid of germinal centers in spleen and lymph nodes (Okkenhaug et al., 2002). This appears to be due to a requirement for p110δ expression in Tfh, rather than B cells, based on subsequent conditional deletion experiments. Mice bearing p110δ^2loxP/2loxP^ alleles were crossed with CD4-Cre to delete p110δ during the double positive thymocyte stage or Ox40-Cre to delete p110δ in peripheral T cells after their initial activation. In both types of mice, PD-1+ CXCR5+ Tfh are decreased 10-fold and 5-fold, respectively with corresponding decreases in the number of germinal center B cells and the number of germinal centers per follicle within lymph nodes. Mechanistically, this correlates with decreased ICOS-induced AKT S473 phosphorylation and reduced expression of factors critical for T cell-dependent B cell help including CD40L, IL-21, IL-4, and c-Maf in T cells lacking p110δ expression (Rolf et al., 2010). A detailed Tfh phenotype has not been reported following T cell conditional deletion of Rictor, however the data from the PI3K deficient mice would predict that Tfh effector cytokines might be reduced.

Despite the controversy surrounding whether p110γ is coupled directly to TCR or CD28, it is clear that loss of p110γ impacts T cell effector function. Both CD4 and CD8 p110γ^−/−^ T cells have defective effector T cell migration *in vivo* that correlates with impaired migratory responses to chemokines *in vitro* (Martin et al., 2008; Thomas et al., 2008). Expression of granzyme B and IFN-γ appears normal in p110γ^−/−^ CD8 effector T cells generated *in vivo* by antigen exposure (Martin et al., 2008). Effector differentiation of p110γ^−/−^ CD4 T cells *in vivo* has not been fully described and impaired footpad swelling induced by LCMV and impaired T-dependent antibody responses in p110γ^−/−^ mice may be related to loss of p110γ expression in cells other that CD4 T cells (Sasaki et al., 2000). Anti CD3/CD28 induced phosphorylation of AKT S473 is decreased in p110γ^−/−^ T cells, suggesting that CD4 effector differentiation may be abnormal in a manner analogous to mTOR or mTORC2 deficient T cells but in light of partial functional redundancy of p110δ activity in such cells, direct measurement of mTOR substrate phosphorylation in response to antigen or anti CD3/CD28 will ultimately need to be performed to address whether the p110γ^−/−^ phenotype is mTOR related.

In contrast to immune dysregulation associated with Treg dysfunction after reducing PI3K activity, enhancing PI3K function by PTEN deletion in the OX40-Cre T cell conditional model not only enhances IFN-γ production *in vitro*, but also results in enhanced Th1 effector function *in vivo* by several measures. PTEN deletion in T cells increases contact hypersensitivity reactions. Adoptively transferred OX40-Cre PTEN floxed OT2 TCR Tg T cells secrete more IL-2 and IFN-γ after activation by infection with attenuated *Listeria* expressing ovalbumin and support better expansion of endogenous ovalbumin-specific CD8 T cells than WT OT2 cells. In addition, adoptively transferred OX40-Cre PTEN floxed OT2 T cells are better at rejecting syngeneic tumor expressing ovalbumin antigen (Soond et al., 2012). These findings are consistent with a robust Th1 response in the presence of increased PI3K/AKT/mTOR activity, but experiments to dissect if any findings were exclusively due to enhanced mTOR activity await experiments culturing PTEN null T cells or treating Ox40-Cre PTEN floxed mice with rapamycin or mTOR kinase inhibitors.

## A model for PI3K/AKT/mTOR regulation of T cell effector function

**Figure 1:**
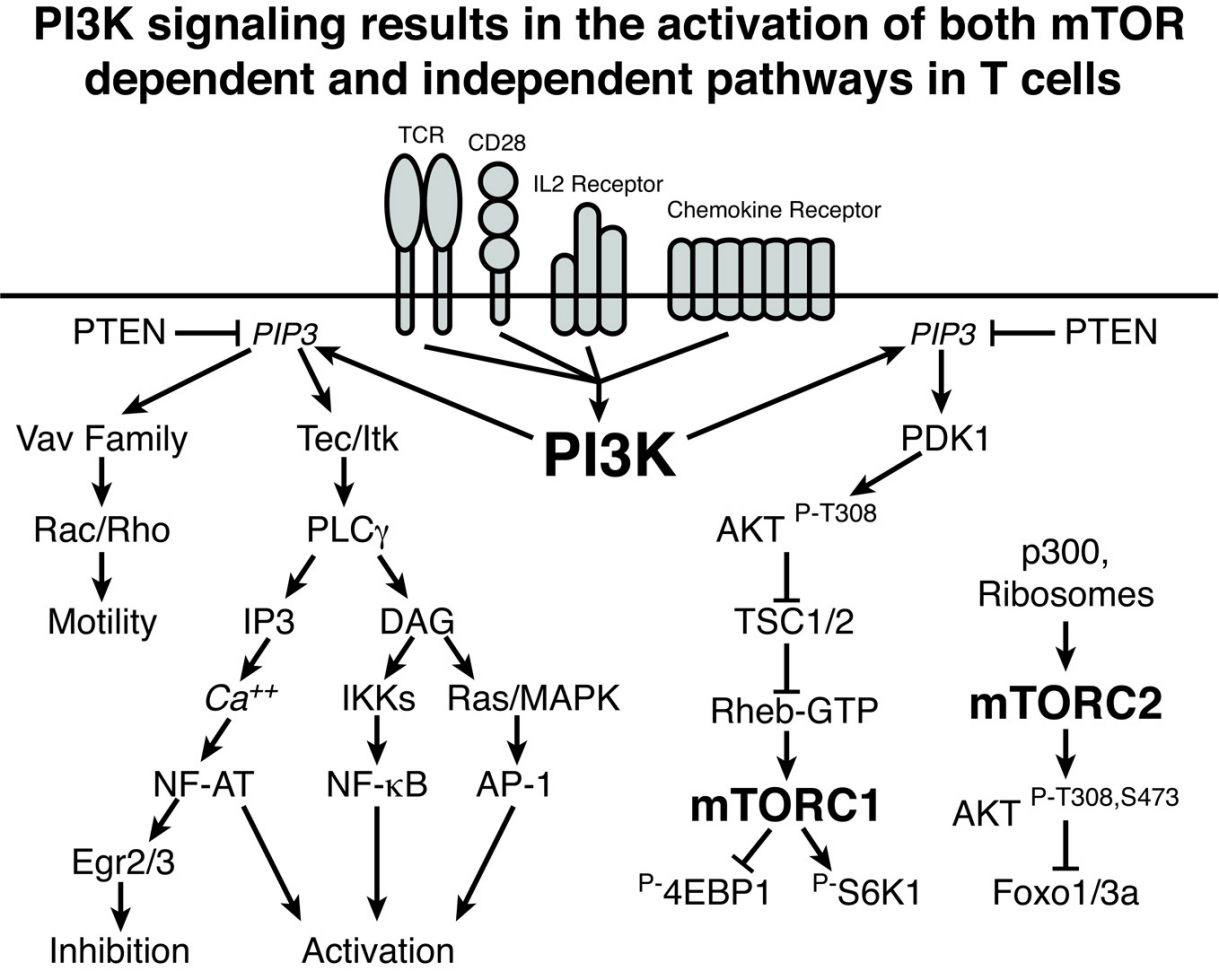
**PI3K signaling results in the activation of both mTOR-dependent and independent pathways in T cells**. A schematic representation of PI3K signaling is shown demonstrating mTOR-independent and mTOR-dependent signaling cascades. Lines with arrows indicate activating signals and lines with bars indicate inhibitory signals. Importantly, this graphic does not include all of the nuanced inputs that are discussed in the text. Rather, these pathways represent connections and not absolute requirements for signaling. For example, while elimination of p110δ or all PI3K regulatory subunits leads to decreased NF-κB, such a deficit can be overcome by the addition of CD28 signaling. Overall, PI3K-induced mTOR-independent signaling can influence both the activation and inhibition of T cells as well as the generation and function of Tregs. The PI3K-induced mTOR-dependent pathways can influence CD4 effector differentiation and function as well as inhibit the generation of Tregs.

In an effort to propose a unifying model to explain the varied phenotypes arising from blockade of the PI3K/AKT/mTOR axis at different points, it is useful to consider experiments that disrupt more proximal and distal signaling events in T cells. Premature interruption of CD3/CD28 signaling or activation by very low doses of cognate peptide in CD4 T cells promotes expression of FoxP3 that is further enhanced by exposure to PI3K inhibitors and rapamycin (Kang et al., 2008; Sauer et al., 2008). Through use of dose responses to selective PI3K inhibitors, Sauer et al. attribute FoxP3 induction to inhibition of PI3Kα and δ upstream of AKT and mTOR. These findings raise a paradox: small molecule inhibitors of PI3K, AKT, and mTOR all result in a similar phenotype, promoting induction of FoxP3 expression following activation of naïve CD4 T cells, but the phenotypes of mice with genetic lesions resulting in loss of T cell PI3K expression demonstrate inefficient Treg production and decreased Treg function. Furthermore, mice with T cell conditional deletion of mTOR phenocopy the small molecule inhibitors and generate Tregs at the expense of effector helper T cells. These observations serve to highlight that there are mTOR-dependent effects of PI3K, mediated by AKT, that are necessary to suppress FoxP3 expression and promote normal effector function and that there are mTOR-independent effects of PI3K, some of which are necessary for normal inducible Treg differentiation. This is shown schematically in Figure 1, and details of the potential mechanisms for this effect are discussed below.

There are several potential mechanisms, currently largely speculative, to explain how loss of PI3K enzymatic activity, with associated decreased mTOR activity, may result in fewer and less functional Tregs. First, PI3K-dependent accumulation of PIP3 results in localization of other families of signaling molecules near the immunologic synapse including Vav guanine nucleotide exchange factors for Ras related GTPases Rac and Rho that control cell motility and Tec family tyrosine kinases that mediate PLCγ activation [reviewed in Cantrell (2001)]. A role for such pathways in T cell activation and effector cytokine expression is well established, but a direct connection between PI3K, Tec, Vav, and Treg function is incompletely characterized. However, disruption of other molecules downstream, including the Bcl-10 and PKCθ components of the NF-κB signaling pathway distal to the Tec family kinases, impairs generation of Tregs (Schmidt-Supprian et al., 2004). Also, Tregs normally have elevated levels of PTEN expression. This is associated with defective phosphorylation of AKT S473 in response to IL-2 stimulation in Tregs as compared to effector T cells and with defective Treg proliferation in response to TCR stimulation in the presence of IL-2 without concurrent CD28 ligation (Bensinger et al., 2004). Conditional deletion of PTEN in T cells does not affect generation of Tregs, the amount of FoxP3 they express, or the suppressive capacity of Tregs, but it does result in enhanced Treg proliferation in response to IL-2 or TCR stimulation (Walsh et al., 2006). Taken together, these data suggest that the Treg defects following loss of PI3K expression might be due to differential sensitivity of Tregs to decreased levels of PIP3 compared to effector cells due to high Treg PTEN expression. Preferential inhibition of Treg proliferation and suppressor cytokine production would be predicted to result in an enhanced ratio of effector T cells to Tregs and autoimmunity in the PI3K deficient mice. Such hypotheses await further experimental evaluation.

Another paradox that arises comparing T cells lacking PI3K with those lacking mTOR is how do PI3K null cells promote some effector function if they are unable to activate mTOR? It is important to remember that other inputs in addition to TCR and CD28-induced PI3K/AKT are able to activate mTOR. In particular, chemokine receptors via p110γ, cellular energy stores by regulating the adenosine monophosphate-dependent kinase (AMPK) pathway, cytoplasmic amino acids via the Ras-related small GTPase Rag proteins, and hypoxia via the hypoxia-induced factor protein regulated in the development of DNA damage response 1 (REDD1)/hypoxia-induced factor 1α (HIF1α) pathway, ERK and RSK downstream of Ras, and phosphatidic acid produced by phospholipase D are potent regulators of mTOR activation [reviewed in Laplante and Sabatini (2012); Waickman and Powell (2012)]. The capacity of these inputs to regulate mTOR activation in T cells is reflected by the ability of 2-deoxyglucose (2-DG) mimicking glucose deprivation, 5-aminoimidazole-4-carboxamide ribonucleoside (AICAR) mimicking cellular energy depletion, and n-acetyl leucine (NALA) mimicking amino acid depletion to result in anergy when present during CD3/CD28 stimulation of T cells, analogous to rapamycin treatment (Zheng et al., 2009). The presence of normal energy, amino acids, and normoxia to promote mTOR activation is therefore consistent with the reported preservation of some mTORC1 activity in the r1ΔT/r2n mice (Deane et al., 2007). This relative increase in mTORC1 activity over mTORC2 activity in these mice supports the observed relative preservation of Th1 cytokine production over Th2 cytokines, analogous to the Rictor deficient T cell mice noted above (Lee et al., 2010; Delgoffe et al., 2011).

Finally, there are two mTOR-dependent pathways that can negatively regulate FoxP3 expression whose loss may explain the increased FoxP3 expression in mTOR null T cells. First, HIF1α is induced by mTORC1 and has been shown to directly bind to FoxP3 protein and promote its ubiquitination and degradation (Dang et al., 2011). Second, the Foxo transcription factors directly bind to and transactivate the FoxP3 promoter, and they are inactivated in an mTORC2-dependent manner following phosphorylation by doubly phosphorylated activated AKT, leading to nuclear export of the Foxos [reviewed in Coffer and Burgering (2004)]. The importance of the latter pathway to Tregs is seen following T cell conditional deletion of Foxo1 and Foxo3. Individually they have overlapping function in T cells to maintain naïve T cells in quiescence, but their simultaneous deletion in T cells results in functional Treg deficiency with early fatal autoimmunity that can be prevented by adoptive transfer of WT Treg (Ouyang et al., 2010). The fact that an increase in Tregs was observed following deletion of mTOR but not in the Rheb and Rictor T cell conditional knockouts suggests that loss of both of these pathways is necessary to result in enhanced generation of FoxP3+ Tregs (Lee et al., 2010; Delgoffe et al., 2011).

## Distal inhibition of the PI3K/AKT/mTOR axis should yield more reliable blockade of T cell mediated pathology

Based on the data and model above, treatment with mTOR inhibitors for immunologic diseases would be predicted to not only suppress immune responses but also potentially promote tolerance by fostering anergy and the induction of Treg. For example, in a mouse model of non-myeloablative peripheral blood stem cell transplantation, we found that treatment with cyclosporine led to eventual graft rejection while treatment with rapamycin led to stable mixed chimerism, even after the rapamycin was stopped. Such engrafted mice had negative mixed lymphocyte reactions to both donor and recipient target cells, indicating tolerance was achieved (Powell et al., 2005). This preclinical data served as the basis for a trial of human transplantation to treat sickle cell anemia with matched sibling donors that specifically avoided use of standard calcineurin inhibitors for graft versus host disease (GVHD) prophylaxis. Patients received alemtuzumab for T cell depletion, low dose total body irradiation, and peripheral blood stem cell transplant with rapamycin for GVHD prophylaxis. Nine of ten patients engrafted with mixed donor chimerism sufficient to abrogate symptoms of sickle cell disease, and patients that have tapered off their immunosuppression have demonstrated stable engraftment, consistent with donor-specific tolerance (Hsieh et al., 2009).

In contrast to the effects of mTOR blockade, loss of PI3K expression in T cells leads to consequences on both Treg and effector T cell function, with the net effect of enhanced or suppressed immune responses *in vivo* strongly dependent on the context. A recent study examined PI3K inhibition as a means of suppressing cardiac allograft rejection (Ying et al., 2012). Transplanting male hearts into female mice results in HY antigen driven chronic allograft rejection that is mitigated if the hearts are transplanted into p110δ^D910A/D910A^ female recipients. Splenocytes from both rejecting WT and non-rejecting p110δ^D910A/D910A^ recipient mice proliferate equally well to HY peptide, however, indicating that the absence of rejection in p110δ^D910A/D910A^ recipients is not due to tolerance. Instead, defective trafficking appears to prevent rejection, as adoptive transfer of HY specific TCR Tg T cells demonstrates reduced localization of the p110δ^D910A/D910A^ HY-specific T cells into male allografts despite normal trafficking of adoptively transferred cells to other tissues. Based on this, the authors tested the selective PI3K p110δ inhibitor IC87114 and found it also prevents rejection by blocking localization of alloreactive cells to the graft but fails to induce tolerance. The inability of proximal PI3K blockade to induce transplant tolerance resembles the inability of cyclosporine to promote engraftment in the stem cell transplant model discussed above and stands in contrast to the tolerance induced by rapamycin in that model.

Interestingly, the capacity for proximal PI3K blockade to potentiate immune function in specific contexts may nicely synergize with the broader ability of PI3K blockade inhibit tumor cell proliferation to make PI3K inhibitors particularly well suited to cancer treatment, reviewed in So and Fruman (2012). To this end, mice were given subcutaneous implants of different syngeneic tumors and then treated with immunotherapy in the presence or absence of ZSTK474, a pan-class I PI3K inhibitor. It was found that this drug both inhibits tumor growth and has the ability to enhance the efficacy of immunotherapy (Marshall et al., 2012). The authors did not assess whether impaired Treg function contributed to tumor clearance, but they demonstrate that inhibition of PI3K is sufficient to suppress DC secretion of IL-10 while permitting secretion of IL-12. This appears to result in enhanced Th1 immunity since neutralization of IFN-γ *in vivo* by blocking antibody abrogated the protection afforded by immunotherapy plus ZSTK474. In this regard, we would predict that mTOR inhibition in the absence of proximal PI3K blockade might also have directs effects on inhibiting tumor growth but would serve to limit development of anti-tumor immunity.

## Summary

By employing genetic analysis it has become clear that in T cells what initially appeared to be a straightforward connection between components of the PI3K signaling cascade via AKT to mTOR is substantially more complex. We have only been able to show a small subset of data published on the topic to illustrate this point and apologize to authors for whom we were not able to include their work. While inhibiting PI3K and mTOR interferes with T cell effector function, what has become clear is that through the inhibition of distal targets in these signaling pathways, selective effects can be achieved. For example, a drug that blocks mTORC2 might be helpful to treat Th2-mediated asthma without blunting Th17 and Th1-mediated antifungal and antibacterial responses. Conversely, an mTORC1 selective drug, unlike rapamycin that can inhibit mTORC2 under prolonged exposure to higher doses, might be helpful in a disease like multiple sclerosis mediated by Th17 and Th1 cells but might permit effective vaccine responses to seasonal influenza by preserving Tfh function. As the PI3K-induced mTOR-independent pathways in T cells become more precisely defined, blockade of nodes distal in the pathway might avoid unintended effects that interfere with function of Treg. Finally, it may turn out that concomitant distal inhibition of both mTOR-dependent and independent pathways will lead to the most robust and precise clinical outcomes.

### Conflict of interest statement

The authors declare that the research was conducted in the absence of any commercial or financial relationships that could be construed as a potential conflict of interest.
